# Activin Receptor-Like Kinase 1 Combined With VEGF-A Affects Migration and Proliferation of Endothelial Cells From Sporadic Human Cerebral AVMs

**DOI:** 10.3389/fncel.2018.00525

**Published:** 2019-01-09

**Authors:** Qiang Hao, Hao Wang, Jun-Lin Lu, Li Ma, Xiao-Lin Chen, Xun Ye, Ya-Hui Zhao, Ming-Tao Li, Yu Chen, Yuan-Li Zhao

**Affiliations:** ^1^Department of Neurosurgery, Beijing Tiantan Hospital, Capital Medical University, Beijing, China; ^2^Department of Neurosurgery, Peking University International Hospital, Peking University, Beijing, China; ^3^China National Clinical Research Center for Neurological Diseases, Beijing, China; ^4^Stroke Center, Beijing Institute for Brain Disorders, Beijing, China; ^5^Beijing Key Laboratory of Translational Medicine for Cerebrovascular Disease, Beijing, China; ^6^Basic Medical Science Department, Capital Medical University, Beijing, China

**Keywords:** cerebral arteriovenous malformation, endothelial cells, morphology, migration, proliferation, activin receptor like kinase 1, VEGF-A

## Abstract

Heterozygous loss of activin receptor-like kinase 1 (Alk1) can lead to hereditary hemorrhagic telangiectasia (HHT), which is a kind of vascular disease characterized by direct connections between arteries and veins with the lacking of capillaries, and develops into arteriovenous malformations (AVMs) in later stage. However, the changes of Alk1 in human sporadic cerebral AVMs (cAVMs) remain unknown. In the present study, we used endothelial cells (ECs) derived from human cAVMs (cAVM-ECs) specimens, to explore the characteristics of cAVM-ECs and the relationship between Alk1 and human sporadic cAVMs. Our data showed that there were obvious morphological changes in cAVM-ECs, and they could trans-differentiate into mesenchyme-like cells easily in a short period. In addition, the abilities of migration of cAVM-ECs were poorer than that in human aortic endothelial cells (HA-ECs). The abilities of proliferation of cAVM-ECs in patients with different ages were lower than HA-ECs. Immunofluorescent staining and Western blot showed that the levels of Alk1 mRNA and protein in the HA-ECs were both higher than that in cAVM-ECs. In addition, the levels of Alk1 mRNA had no significant differences between different ages in cAVM-ECs groups. The levels of VEGF-A mRNA in the cAVM were higher than HA-ECs. Besides, levels of VEGF-A mRNA expression were lower in older cAVM patients. Therefore, we conclude that Alk1 might induce the formation of sporadic human cAVMs through affecting migration and proliferation of endothelial cells combined with VEGF-A.

## Introduction

Cerebral arteriovenous malformations are vascular disorders characterized by an anomalous tangle of nidus in vessels, which are characterized by direct connections between arteries and veins with the lacking of capillaries. Hemorrhage is one of the most serious complications (da Costa et al., 2009; Lawton et al., 2015). Therefore, the most important therapeutic strategy is to prevent the rupture of cAVMs. Current clinical treatments include surgical resection, radiotherapy, and embolization. However, all of these treatments may bring high risks of morbidity, leading to serious complications (da Costa et al., 2009). For example, surgical resection may induce neural functional deficits when cAVMs are located in the functional areas; the intervention of radiosurgery may be restricted by sizes of cAVMs (Wang et al., 2016). However, embolization is a minimally invasive treatment, which may be restricted when cAVMs are scattered and/or feeded by small feeding arteries (Sammons et al., 2011). Therefore, biological therapeutic method may be a promising option for cAVMs. Before biological therapies are developed, we should fully understand the pathophysiology and molecular mechanisms of cAVMs. As an important component of vessels in cAVMs, the endothelium has great theoretical potential as a therapeutic target.

Based on the researches of histological properties of cAVMs in patients with hereditary hemorrhagic telangiectasia (HHT), we speculate that the development of cAVMs originate from a focal dilation of a post capillary venues, leading to arteriole dilation progression and subsequent loss of intervening capillaries (Braverman et al., 1990). In previous studies, sub-dermal arteriovenous malformations (AVMs) are induced in wounds of activin receptor-like kinase 1 (Alk1)-mutant mice, which appeared in the form of both arteries and veins elongation followed by arterial-venous connections *de novo* (Kim et al., 2011; Laux et al., 2013; Garrido-Martin et al., 2014). Although these solid findings are confirmed by the longitudinal imaging of vascular growth, they still remained unclear in cellular level. Therefore, abnormal behaviors of endothelial cells (ECs) may cause AVMs and need to be elucidated in the sporadic patients with cAVMs. Considering the possible relationship between Alk1 function and cAVMs development, we hypothesized that Alk1 may play an important role in the formation of cAVMs by trans-differentiation and changes of ability of migration and proliferation in ECs derived from cAVMs.

Therefore, in this study, we isolated ECs from sporadic human cAVMs patients, to explore the potential mechanisms of occurrence and development of cAVMs in sporadic patients and get a better understanding *in vitro*.

## Materials and Methods

### Isolation of ECs

Human surgical specimens were obtained in accordance with The Human Subject Review Committee of Tian Tan Hospital. cAVM-ECs were obtained from four males and four females, aged from 8 to 51 years old (average of 29.6 ± 14.55). Patients who involved in this study had not adopted gamma knife irradiation or embolization, and information was described in the Table 1. cAVM-ECs were isolated and selected by Flow cytometer, and identified by detecting ECs special markers (CD31 and CD34). In addition, their functional properties were detected by LDL-uptake and tube formation. All of cAVM-ECs used in this experiment were passaged to the forth generation at most. Human aortic endothelial cells (HA-ECs) were gained from ScienCell Company (ScienCell, Cat No. 6100, United States) and used as control ECs.

**Table 1:** Clinical data of cerebral arteriovenous malformations patients who underwent microsurgical cAVMs resection.

Patient No.	Age (years)	Sex	Hemorrhage	cAVM size (cm)	Spetzler-Martin grade	Radiosurgery
1	14	Female	Yes	3	3	No
2	32	Male	No	4	4	No
3	51	Female	No	4	4	No
4	29	Female	Yes	5	5	No
5	17	Female	No	4	4	No
6	44	Male	Yes	6	5	No
7	8	Male	Yes	3	3	No
8	42	Male	No	5	4	No

*The cAVMs endothelial cells (cAVMs-ECs) used in this experiment were derived from them.*

### Immunocytochemistry

cAVM-ECs and HA-ECs were fixed in 4% paraformaldehyde for 20 min at RT, then blocked in 5% goat serum and incubated at 4°C with the following primary antibody: Rabbit polyclonal anti-human Alk1 (1:200, Cat No. ab68703, Abcam, United States). The slides were then incubated with the appropriate secondary antibody: Anti-Rabbit IgG H&L (Alexa Fluor^®^488) (1:200, Cat No. ab150077, Abcam, United States) for 1 h. Coverslips with immunostained cells were mounted on glass slides in Vectashield mounting medium containing DAPI (Vector Laboratories, Cat No. H1200, United States). Immunostained cells were counted randomly from five fields under a Nikon Eclipse microscope.

### Cell Migration Assay

The ability of ECs migration was detected by scratch test. cAVM-ECs and HA-ECs were seeded into a 12-well plate at the concentration of 0.5 × 10^5^ cells per well, respectively. When cells contacted with each other, they were scratched vertically with a 100 μl-pipette tip. Then, cells were washed twice with PBS and placed in serum-free culture medium. After 6 and 12 h, distances between the two sides of cells were measured under an invert phase-contrast microscope in five random fields, respectively. Each migration test was run in triplicate.

### Cell Proliferation Assay

cAVM-ECs from eight patients were successfully passaged to the forth generation. To detect the proliferative ability of these cells, we seeded different patients’ cAVM-ECs at the density of 0.1 × 10^5^ per well, and the cells were cultured for 8 days. Then the cAVM-ECs were digested and counted. HA-ECs were used as control.

### Western Blot

For preparation of total protein lysates, cells were lysed in cold RIPA buffer and centrifuged at 13,000 ×*g* for 5 min at 4°C. Protein lysates were subjected to 10% SDS-PAGE gels, and then were transferred onto PVDF membranes. After blocking with 5% non-fat dried milk for 2 h at RT, membranes were incubated with the primary antibodies overnight at 4°C: Primary antibodies against Alk1 (1:1,000, Cell Signaling Technology, Cat No. 31278, United States) and GAPDH (1:5,000, Cell Signaling Technology, Cat No. 51332, United States), each primary antibody was diluted in blocking buffer and then incubated overnight at 4°C. The membranes were washed three times and incubated with horseradish peroxidase (HRP)-linked secondary antibody at RT for 1 h. Membranes were washed three times with TBST at RT. Protein bands were visualized on X-ray film. GAPDH was used as a loading control.

### Quantitative Real-Time PCR

According to the manufacturer’s protocol, total RNAs were isolated from EC using TRIzol reagent (Invitrogen). cDNAs were synthesized by reverse transcription using SuperScript III First-Strand Synthesis System kit (Invitrogen). TaqMan Gene Expression Assays (Applied Biosystems) was used for qPCR to quantify relative expression of each gene using Mx3000P QPCR System (Agilent Technologies). Predesigned qPCR primers: Alk1 (forward): 5′-CCACTCATTCCTCCTGGGTA-3′, Alk1 (reverse): 5′-ACTTCCTGACTAGGGGAGGAGTAG-3′; VEGF (forward): 5′-CCCACGAAGTGGTGAAGTTCA-3′, VEGF (reverse): 5′-CCACCAGGGTCTCGATGG-3′; GAPDH (forward): 5′-GATGGTGAAGGTCGGAGTGAAC-3′, GAPDH (reverse): 5′-GTCATTGATGGCGACGATGT-3′. RNase free dH_2_O was used as the negative control reaction.

### Statistical Analysis

All data were presented as Mean ± SD and subjected to statistical analysis using GraphPad Prism software 6.0 (United States). Student’s *t*-test was used for group comparisons between two groups. When there were more than two groups, the one-factor ANOVA (ANOVA) followed by Tukey’s *post-hoc* test was used for comparisons between groups. Statistical significance was defined at *p* < 0.05.

## Results

### Isolated ECs From Human cAVM (cAVM-ECs)

**FIGURE 1:**
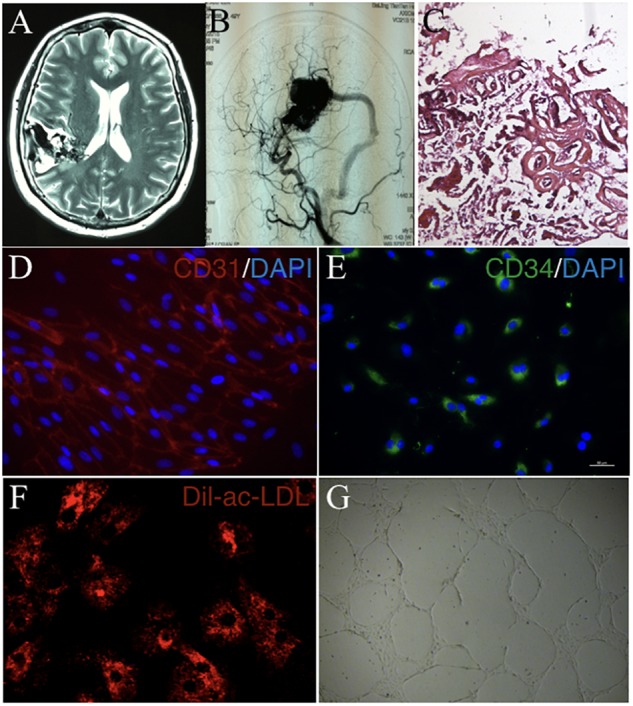
The general characteristics of cAVM and cAVM-ECs. **(A)** T2WI of MRI imaging; **(B)** Digital subtraction angiography (DSA) showed cerebral middle artery provided blood for the cAVM nidus as a feeder artery, which connected to the sigmoid sinus through thick draining vessel; **(C)** Photomicrograph of cAVM with deformed vascular wall, and the nidus of the cAVM had many collagen fibers, lacking of smooth muscle and elastic fibers; **(D,E)** Immunostaining of ECs showed special markers CD31 and CD34 expressed in cAVM-ECs; **(F)** LDL uptake assay of cAVM-ECs; **(G)** Tube formation of cAVM-ECs. Scale bar = 200 μm.

cAVMs were characterized by conglomerate of tortuous vessels, including feeding arteries and draining veins. We found cluttered vessels flow void in the right temporal-parental area in T2WI of MRI imaging (Figure 1A). Digital subtraction angiography (DSA) results showed that cerebral middle artery provided blood for the cAVM nidus as feeder artery, which was connected with the sigmoid sinus through thick draining vessel (Figure 1B). Under the operational microscope (Carl Zeiss, Pentero 800, United States), we found abnormal vessels nidus occupied on the brain surface scrabbly (Figure 1C). In addition, immunostaining results demonstrated that cAVM-ECs expressed typical EC markers, such as CD31 and CD34 (Figures 1D,E). These cells derived from cAVMs also possessed the functional properties of general ECs, including the ability of Dil-Ac-LDL uptake and tube formation on 3D matrigel (Figures 1F,G). Therefore, the isolated cAVM-ECs not only have the cellular characteristics in morphology and special markers of ECs, but also have the common functional properties of ECs (Hao et al., 2018).

### Morphological Characteristics of cAVM-ECs

**FIGURE 2:**
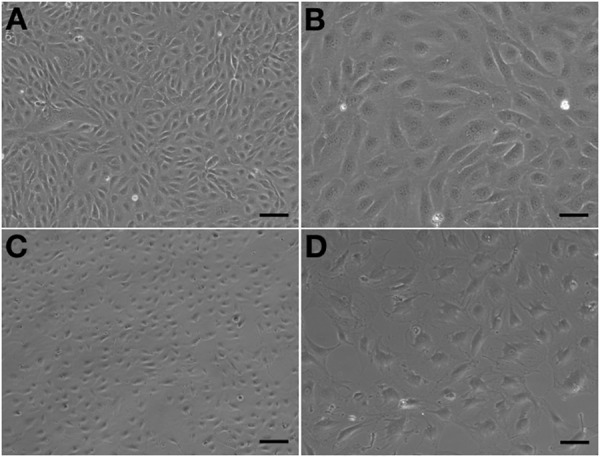
Morphology of human aortic endothelial cells (HA-ECs) and cAVM-ECs. **(A,B)** Morphology of HA-ECs under microscope in **(A)** 40×, Scale bar = 100 μm; **(B)** 100×, Scale bar = 50 μm, showed classical round borders and cobblestone appearance; **(C,D)** showed irregular borders and flatty bodies with smaller nuclei, **(C)** 40×, Scale bar = 100 μm; **(D)** 100×, Scale bar = 50 μm.

**FIGURE 3:**
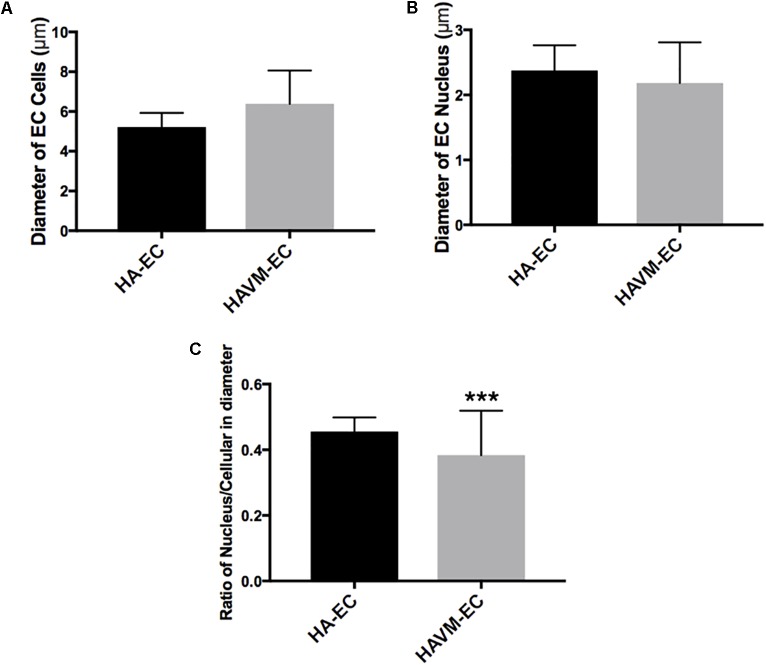
Changes of morphology in cAVM-ECs. **(A)** Average diameters of body in cAVM-ECs and HA-ECs, (6.391 ± 0.5283) μm in cAVM-ECs, were larger than that (5.222 ± 0.2224) μm in HA-ECs (*p* > 0.05); **(B)** The average diameters of nucleus in cAVM-ECs (2.183 ± 0.1981) μm, were smaller than that (2.377 ± 0.1226) μm in HA-ECs (*p* > 0.05); **(C)** Ratios of nucleus to cellular in cAVM-ECs was (0.3437 ± 0.02426), and that in HA-ECs was (0.4557 ± 0.01356), and there was a significant difference between them (*p* < 0.05).

Observed under a phase-contrast microscope, the HA-ECs had classical round borders and cobblestone appearances (Figures 2A,B). When cAVM-ECs contacted with each other, they showed irregular borders and flatty bodies (Figures 2C,D). Compared to HA-ECs, these isolated cells showed rough edges and had no obvious gloss. In addition, the average diameter of cAVM-ECs body was 6.391 ± 0.5283 μm, which was larger than that in HA-ECs (5.222 ± 0.2224 μm) (*p* > 0.05); the average diameter of nucleus of cAVM-ECs was 2.183 ± 0.1981 μm, which was smaller than that in HA-ECs (2.377 ± 0.1226 μm) (*p* > 0.05). There was no significant difference between cAVM-ECs and HA-ECs. However, the ratio of nucleus to cellular in diameter of cAVM-ECs (0.3437 ± 0.02426) was smaller than that in HA-ECs (0.4557 ± 0.01356), and there was significant difference (*p* < 0.05) (Figure 3). These results showed that obvious changes existed in the morphology of cAVM-ECs.

### Trans-Differentiation Into Mesenchyme-Like Cells in a Short Period

**FIGURE 4:**
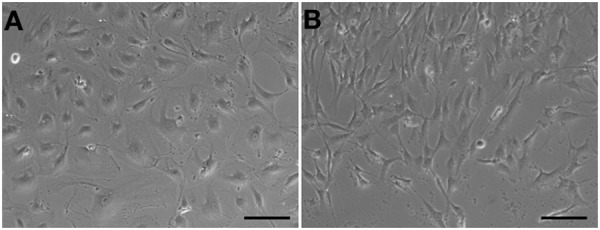
cAVM-ECs trans-differentiated into mesenchyme-like cells in a short period. **(A)** HA-ECs kept the general morphology of ECs when passaged to 7th–8th population without morphological change; **(B)** The morphology of cultured cAVM-ECs changed into spindle-like cells after passaged to the forth generation.

Human aortic endothelial cells could be passaged to seventh or eighth population without morphological changes (Figure 4A). However, compared to HA-ECs, the morphology of cAVM-ECs changed into spindle-like cells after passaged to the forth generation (Figure 4B). These results demonstrate that the cAVM-ECs can not maintain the characteristics of morphology for a long time, and easily trans-differentiate into mesenchyme-like cells in a short period.

### Poor Ability of Migration in cAVM-ECs

**FIGURE 5:**
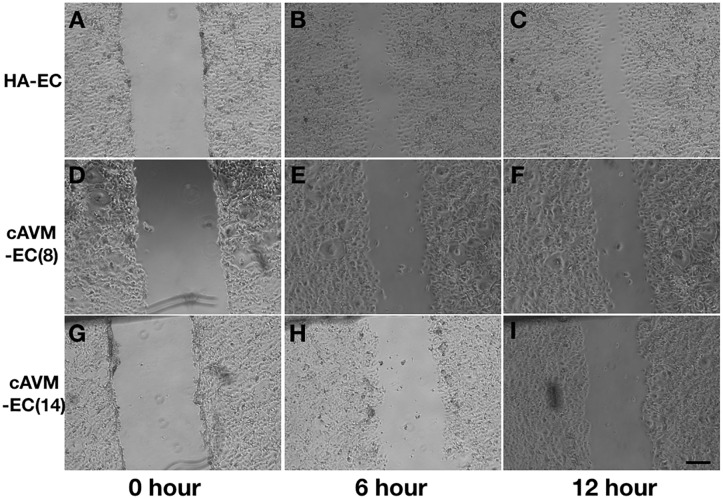
Scratch assay in cAVM-ECs and HA-ECs. When HA-ECs and cAVM-ECs (derived from 8- and 14-year-old patients) were seeded in the 24-well plate, we detected the ability of migration among three groups at 6 and 12 h after seeding, respectively. **(A–C)** Scratch assay in HA-ECs, **(A)** 0 h after Scratched, **(B)** 6 h after Scratched, **(C)** 12 h after Scratched; **(D–F)** Scratch assay in cAVM-ECs (derived from 8-year-old patient), **(D)** 0 h after Scratched, **(E)** 6 h after Scratched, **(F)** 12 h after Scratched; **(G–I)** Scratch assay in cAVM-ECs (derived from 14-year-old patient), **(G)** 0 h after Scratched, **(H)** 6 h after Scratched, **(I)** 12 h after Scratched; Scale bar = 100 μm.

**FIGURE 6:**
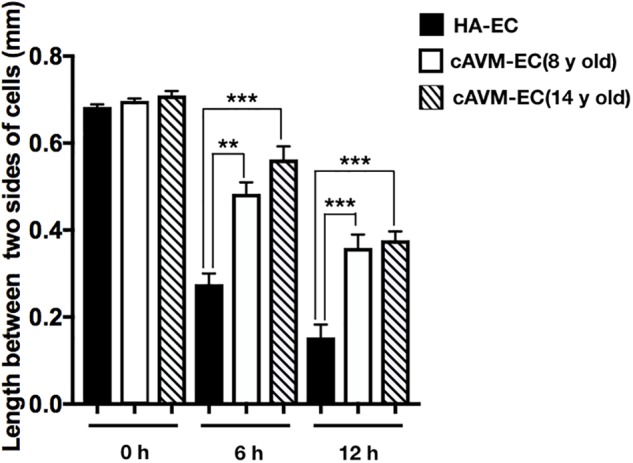
Statistical analysis of scratch assay in cAVM-ECs and HA-ECs. After 6 h, the distances between two sides of scratch in HA-ECs were (0.276 ± 0.024) mm, (0.483 ± 0.026) mm in cAVM-ECs of 8-year-old, and (0.563 ± 0.030) mm in cAVM-ECs of 14-year-old (all ^∗^*p* < 0.05). After 12 h, the distances between two sides of scratch in HA-ECs were (0.153 ± 0.029) mm, (0.359 ± 0.030) mm in cAVM-ECs of 8-year-old, (0.376 ± 0.021) mm in and cAVM-ECs of 14-year-old (all ^∗^*p* < 0.05). Besides, the distances between two sides of scratch in cAVM of 8- and 14-year-old patients had no significant difference.

After HA-ECs and cAVM-ECs (derived from 8- and 14-year-old patients) were seeded in the 24-well plate, we analyzed the ability of migration among three groups in 6 and 12 h, respectively. Because of limitation of cAVM-ECs sources, we just detected cAVM-ECs from 8- to 14-year-old patients. The distances between two sides of scratch in HA-ECs were shorter than that in the other two cAVM-ECs groups, measured, respectively, at 6 and 12 h after seeded. 6 h later, the distances between two sides of scratch in HA-ECs were 0.276 ± 0.024 and 0.483 ± 0.026 mm in cAVM-ECs of 8-year-olds, and 0.563 ± 0.030 mm in cAVM-ECs of 14-year-olds (all^∗^*p* < 0.05). 12 h later, the distances between two sides of scratch in HA-ECs were 0.153 ± 0.029 mm, 0.359 ± 0.030 mm in cAVM-ECs of 8-year-old, and 0.376 ± 0.021 mm in cAVM-ECs of 14-year-old (all ^∗^*p* < 0.05). In addition, the distances in cAVM of 8- and 14-year-old patients had no significant difference (Figures 5, 6). These results demonstrate that ability of migration in the cAVM-ECs groups decreases significantly compared to HA-ECs group.

### Proliferative Ability of cAVM-ECs Derived From Different Age Patients

**FIGURE 7:**
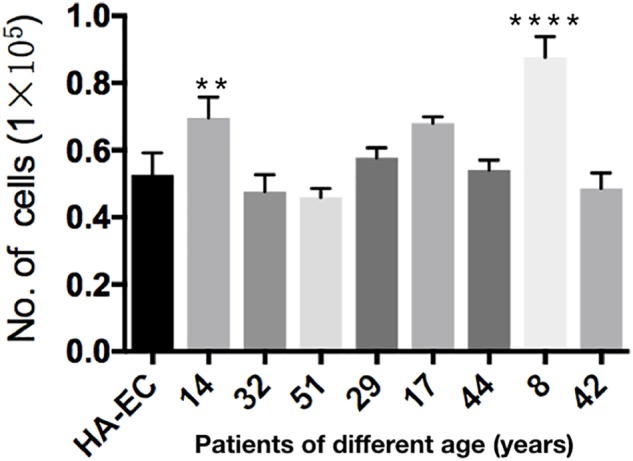
Proliferative ability of HA-ECs and cAVM-ECs with different ages. HA-ECs and cAVM-ECs derived from eight patients were successfully passaged to the forth generation. The results showed that the number of HA-ECs was (0.527 ± 0.065) million, which was less than that in cAVM-ECs from (patient 8-year-old) (0.877 ± 0.06) million and cAVM-ECs from (patient 14-year-old) (0.697 ± 0.061) (all ^∗^*p* < 0.05).

Human aortic endothelia cells and cAVM-ECs from eight patients were successfully passaged to the forth generation. To detect the proliferative ability of these cells, we seeded HA-ECs and cAVM-ECs derived from different age patients at the density of 0.1 × 10^5^ per well. Cultured for 8 days later, all of the ECs were digested and counted. The results showed that the number of HA-ECs was 0.527 ± 0.065 million and less than that in cAVM-ECs (0.877 ± 0.06 million in 8-year-old patient and 0.697 ± 0.061 million in 14-year-old patient) (all ^∗^*p* < 0.05). In addition, there were no significant differences in other patients groups. Besides, the number of cAVM-ECs in 8-year-old patient was higher than that in all the other groups (^∗^*p* < 0.05) (Figure 7).

### Lower Expression of Alk1 in cAVM-ECs

**FIGURE 8:**
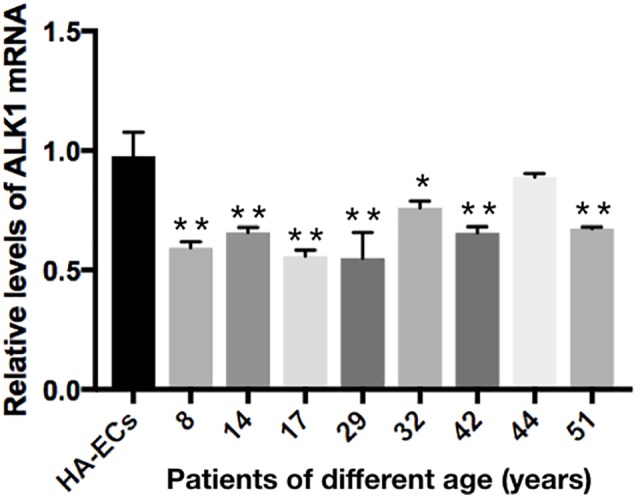
mRNA expression of gene Alk1 in HA-ECs and cAVM-ECs in patients with different ages. Levels of VEGF-A mRNA expression (0.986 ± 0.134) in the HA-ECs were lower than that in cAVM-ECs (8 years) (4.183 ± 0.238) and cAVM-ECs (14 years) (3.834 ± 0.451) (all ^∗^*p* < 0.05). In addition, the levels of VEGF-A mRNA expression were (2.92 ± 0.58) in cAVM-ECs (17 years), (3.04 ± 0.79) in cAVM-ECs (29 years), (3.08 ± 0.37) in cAVM-ECs (32 years), (2.08 ± 0.28) in cAVM-ECs (42 years), (1.97 ± 0.24) in cAVM-ECs (44 years), and (1.97 ± 0.25) in cAVM-ECs (51 years), all of that were higher than that (0.986 ± 0.134) in HA-ECs (all ^∗^*p* < 0.05).

**FIGURE 9:**
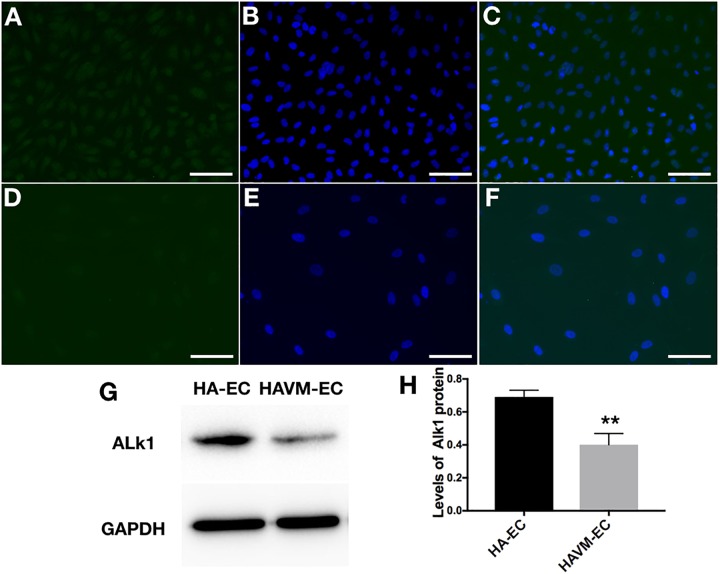
Expression of Alk1 proteins in HA-ECs and cAVM-ECs. **(A–D)** Immunofluorescent staining to detect the Alk1 protein expressed in HA-ECs and cAVM-ECs. **(A–C)** In HA-ECs, **(A)** Alk1 protein was stained by Green fluorescent, **(B)** nuclei was stained by DAPI in Green fluorescent; **(C)** merge; **(D–F)** In cAVM-ECs, **(D)** Alk1 protein was stained by Green fluorescent, **(E)** nuclei was stained by DAPI in Green fluorescent; the fluorescence intensity in cAVM-ECs was obviously lower than in HA-ECs; **(F)** merge. Scale bar = 200 μm; **(G)** Detect the expression of Alk1 by Western blot, **(H)** expression of Alk1 proteins in the HA-ECs cultures were significantly higher than that in cAVM-ECs *in vitro* (^∗^*p* < 0.05).

The levels of Alk1 mRNA were tested by qRT-PCR. Results showed that the levels of expression of Alk1 mRNA were 0.59 ± 0.03 in cAVM-ECs (8 years), 0.66 ± 0.02 in cAVM-ECs (14 years), 0.66 ± 0.02 in cAVM-ECs (17 years), 0.55 ± 0.1 in cAVM-ECs (29 years), 0.76 ± 0.03 in cAVM-ECs (32 years), 0.66 ± 0.02 in cAVM-ECs (42 years), and 0.67 ± 0.01 in cAVM-ECs (51 years), and all of them were lower than that in HA-ECs (0.98 ± 0.1) (all ^∗^*p* < 0.05). In addition, there were no significant differences between the different cAVM-ECs groups. The levels of Alk1 mRNA in cAVM-ECs (44 years) (0.89 ± 0.01) was lower than that in HA-ECs (0.96 ± 0.1), however, there was no significant difference between them (Figure 8). In addition, immunofluorescent staining showed that HA-ECs and cAVM-ECs were Alk1-positive in Cytoplasm. However, the fluorescence intensity in cAVM-ECs was obviously lower than that in HA-ECs (Figures 9A–F). Besides, due to the limitation of the cAVM-ECs sources, we combined all of the cAVM-ECs groups to detect the average levels of Alk1 expression. Western blot results also confirmed that the levels of Alk1 proteins in the HA-ECs were higher than that in cAVM-ECs (^∗^*p* < 0.05) (Figures 9G,H).

### Increasing Expression of VEGF-A in cAVM-ECs

**FIGURE 10:**
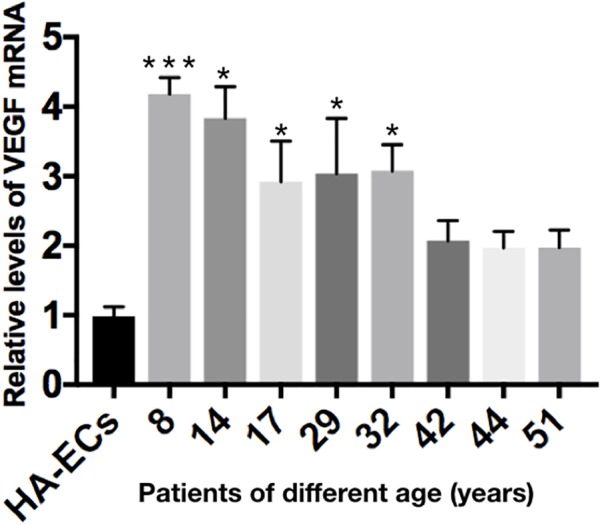
mRNA expression of gene VEGF in HA-ECs and cAVM-ECs with different ages. Level of VEGF-A mRNA expression in the HA-ECs (0.98 ± 0.13) was lower than that in cAVM-ECs (8 years) (4.183 ± 0.238) and cAVM-ECs (14 years) (3.834 ± 0.451) (all ^∗^*p* < 0.05). Level of VEGF-A mRNA expression in cAVM-ECs (17 years) was (2.92 ± 0.58), in cAVM-ECs (29 years) was (3.04 ± 0.79), in cAVM-ECs (32 years) was (3.08 ± 0.37), in cAVM-ECs (42 years) was (2.08 ± 0.28), in cAVM-ECs (44 years) was (1.97 ± 0.24), and in cAVM-ECs (51 years) was (1.97 ± 0.25), all of that were higher than that in the HA-ECs (0.98 ± 0.13) (all ^∗^*p* < 0.05).

The levels of VEGF-A gene expression were examined by qRT-PCR analysis. Results showed the levels of VEGF-A mRNA were 0.98 ± 0.13 in the HA-ECs, which was lower than that in cAVM-ECs (8 years) (4.183 ± 0.238) and cAVM-ECs (14 years) (3.834 ± 0.451) (all ^∗^*p* < 0.05). In addition, the levels of VEGF-A mRNA were 2.92 ± 0.58 in cAVM-ECs (17 years), 3.04 ± 0.79 in cAVM-ECs (29 years), 3.08 ± 0.37 in cAVM-ECs (32 years), 2.08 ± 0.28 in cAVM-ECs (42 years), 1.97 ± 0.24 in cAVM-ECs (44 years), and 1.97 ± 0.25 in cAVM-ECs (51 years), which all of those were higher than that in the HA-ECs (0.98 ± 0.13) (all ^∗^*p* < 0.05). These results showed that the levels of VEGF-A in cAVM-ECs were higher than that in HA-ECs. In addition, the levels of VEGF-A in the older patients with cAVM were lower than that in the younger patients (Figure 10).

## Discussion

The isolated human cAVM-ECs owned the general characteristics of ECs, such as cobblestone-like morphology, the special markers of ECs (CD31 and CD34), and the functional properties of LDL intake and tube formation. However, compared with normal HA-ECs, we found some changes of AVM-ECs in morphology, such as irregular borders, smaller nucleus and bigger cell bodies, all of which might induce functional changes of these cells. For instance, in our experiment, we found that AVM-ECs could differentiate into mesenchyme-like cells when passaged into forth generation. In addition, cAVM-ECs proliferated faster and migrated more slowly than that in HA-ECs. These results demonstrate that ECs play an important role in the occurrence and development in sporadic human cAVMs.

In the scratch assay, we found that the speeds of migration in cAVM-ECs were obviously lower than that in HA-ECs. In addition, we detected the levels of Alk1 expression in cAVM-ECs and HA-ECs, using immunofluorescent staining and qRT-PCR, respectively. The results showed that the density of fluorescent of Alk1 positive cells in cAVM-ECs was lower than that in HA-ECs. In addition, levels of Alk1 proteins expression in HA-ECs were higher than that in cAVM-ECs, detected by Western blot assay. Besides, qRT-PCR results were in accordance with Western blot, and there were no significant differences between the different cAVM-ECs groups. Lower levels of Alk1 expression might inhibit the ability of cAVM-ECs migration. Rochon et al. reported, the main role of Alk1-mutant could not alter the arterial EC proliferation, but could alter the movement of arterial ECs in vessels. In Alk1-mutant ECs, directed migration of ECs could be impaired (Rochon et al., 2016). Therefore we speculate that decreased Alk1 expression results in poorer ability of migration in sporadic cAVM-ECs, and Alk1 plays an important role in the formation of cAVMs.

In the proliferation assay, we compared the proliferative ability between the HA-ECs group and cAVM-ECs with different ages groups. The results showed that cAVM-ECs proliferated faster than HA-ECs. We also detected the levels of VEGF-A expression in HA-ECs and cAVM-ECs with different ages using qRT-PCR. We found that levels of VEGF-A expression in cAVM-ECs were higher than that in HA-ECs. VEGF-A did not only stimulate endothelial migration, but also enhanced the ability of proliferation (Eichmann and Simons, 2012). This conclusion was partly in accordance with our results. However, Pro Gerhardt pointed that neighboring cells contributed to elongation and stability of the new vessel (stalk cells), when some of ECs formed polarized filopodia protrusions and acquire the leading tip position in the nascent sprout (tip cells) (Gerhardt et al., 2003).

In our previous research, we found that the Alk1-mutant mice would not form cAVMs without local VEGF over-expression. Hence, we thought that down-regulation of Alk1 expression combined with the up-regulation of VEGF, could accurately reflect the formation development of cAVMs in the sporadic patients. Actually, we found that the levels of VEGF expression were higher in the sporadic patients than that in normal control in another experiment, detected by ELISA Testing blood samples. The down-regulation of Alk1 expression might influent the ability of migration in ECs, while over-expression of VEGF might enhance the proliferative ability. Both of which could induce the formation of an abnormal vessel nidus. In addition, high doses of VEGF injected in the selected cerebral area of the Alk1-mutant mice could lead to cerebral hemorrhage. Professor Park and Martin reported previously, regional or tissue-specific conditional gene deletion of either Eng or Alk1 could produce AVMs when *de novo* angiogenesis was stimulated by VEGF in mice (Park et al., 2009; Garrido-Martin et al., 2014).

In addition to the above-mentioned, products of ENG and MADH4 were also the receptors or signaling molecules of the TGFβ/BMPs pathway (Urness et al., 2000; Sorensen et al., 2003). They might affect proliferation, differentiation, migration and extracellular matrix formations of ECs, which all played a critical role in the proper development of the blood vessels. Alk1 might promote cell migration and proliferations collaborated with ENG.

In order to further explore the further mechanism of cAVMs formation and development in sporadic patients, we should optimize the methods of isolation and culturing to increase the yield of cAVM-ECs. And the anti-VEGF treatment may be a promising option to treat AVMs. In the future, VEGF might be neutralized to reduce their levels, and to detect whether it could repair the morphology and reduce the rate of hemorrhage induced by cAVMs.

## Conclusion

Alk1 might induce the formation of sporadic human cAVMs through affecting migration and proliferation of ECs, combined with VEGF-A.

## Ethics Statement

This experiment was approved by the Beijing Tian Tan Hospital Ethics Committee (Beijing, China).

## Author Contributions

Y-LZ designed the whole experiments and QH participated in the whole proceed. J-LL, HW, X-LC, LM, XY, M-TL, Y-HZ, and YC took part in the collection of AVM tissues and discussion.

## Conflict of Interest Statement

The authors declare that the research was conducted in the absence of any commercial or financial relationships that could be construed as a potential conflict of interest.
